# ShenNongDiet: a medicine diet recommendation system based on graph-semantic hybrid retrieval and evidence constraint generation

**DOI:** 10.1186/s13020-026-01505-x

**Published:** 2026-08-26

**Authors:** Xiangying Yan, Zhuang Guo, Na Lin, Yanqiong Zhang

**Affiliations:** State Key Laboratory for Quality Ensurance and Sustainable Use of Dao-Di Herbs, Institute of Chinese Materia Medica, China Academy of Chinese Medical Sciences, No. 16, Nanxiaojie, Dongzhimennei, Beijing, 100700 China

**Keywords:** Traditional Chinese medicine diet, Knowledge graph, Retrieval-augmented generation, LoRA fine-tuning, Evidence constraints

## Abstract

**Background:**

Medicinal diet, a core practice in Traditional Chinese medicine (TCM) rooted in the principle of “medicine and food homology”, offers potential for chronic disease management and sub-health regulation. However, the broader adoption is constrained by fragmented knowledge, poor personalization, and safety risks arising from improper ingredient combinations or constitution-therapy mismatches.

**Objective:**

We developed and validated ShenNongDiet, a knowledge graph (KG) and retrieval-augmented generation (RAG) system for TCM dietary recommendation that explicitly models constitution suitability and ingredient contraindications.

**Methods:**

A Neo4j KG was constructed from the “*Guoyi Jinghua Yao Shan*” book series encompassing 3376 medicinal diets, 2110 ingredients, 1176 effects, 1122 population groups, 1078 symptoms, 459 diseases, 38,669 relationships. The system integrates dual-pathway retrieval (symbolic reasoning from KG + dense vector search using Qwen3-embedding-8B), RAG-based evidence packaging, and Low-Rank Adaptation (LoRA) fine-tuning of Qwen3-8B on 8509 evidence-constrained instruction samples to reduce the risk of the model generating unsupported content. A multi-scenario test set was constructed to evaluate system effectiveness and robustness.

**Results:**

ShenNongDiet achieved Hit@1, Hit@3, and Hit@5 of 84.00%, 90.00%, and 92.67% respectively, with a Mean Reciprocal Rank (MRR) of 0.8778. RAGAs evaluation yielded a faithfulness score of 0.831 and context recall of 0.526. Expert human evaluation further confirmed the system’s strong performance in recommendation accuracy, evidence relevance, and generation faithfulness. Ablation experiments confirmed that the hybrid architecture significantly outperformed every single retrieval channel (*P* < 0.001), validating the complementary benefit of the symbolic and semantic retrieval.

**Conclusion:**

This study systematically constructs an explicit-semantics knowledge model of TCM medicinal diet and integrates KG reasoning with language model generation under evidence constraints. ShenNongDiet offers an expandable and safety-constrained solution for intelligent dietary services, facilitating the digital preservation of traditional dietary culture and artificial intelligence-driven personalized health management.

**Supplementary Information:**

The online version contains supplementary material available at 10.1186/s13020-026-01505-x.

## Introduction

Medicinal diet, a core practice in Traditional Chinese Medicine (TCM) rooted in the principle of “medicine and food homology”, is also referred to as “medicated diet” (Yaoshan) by the World Health Organization (WHO). It has long been used for disease prevention, post-treatment recovery, and chronic disease management [1, 2]. However, a growing number of consumers now search for and select medicinal diet information on their own. Unlike the medication process in medical institutions where prescriptions are reviewed by pharmacists, consumers often choose medicinal diet without professional syndrome differentiation guidance or contraindication verification, which may pose potential health risks [3]. Two additional problems compound this situation. First, the semantic gap between everyday language and standard TCM terminology makes it difficult for consumers to accurately retrieve medicinal diet information [4]. Second, most publicly available information lacks clear labeling of key safety aspects such as applicable populations and contraindications [5]. In high-risk scenarios such as pregnancy, pediatric use, or allergic constitutions, there are no predictive tools comparable to drug interaction warnings [6]. These problems constitute important research gaps in health informatics and clinical decision support.

Recent advances in large language models (LLMs) and knowledge graphs (KGs) offer technical support for the recommendation systems of medicinal diets. Li et al. developed the TCM-DS model based on the DeepSeek-R1 model, achieving the recommendation of medicinal diet based on symptoms and constitution through Low-Rank Adaptation (LoRA) fine-tuning and retrieval-augmented generation (RAG) technology, with a recommendation accuracy of 0.9924. However, its knowledge base contains only 288 recipes and 484 symptoms, and no explicit association between medicinal diets and population-specific contraindications has been established [7]. Sha et al. introduced Yaoshi-RAG, a system that leverages a medicinal diet KG containing 25,000 entities and 29,000 triples, and enhances LLMs through uncertainty reasoning and path retrieval, achieving a Hits@1 of 84.2%. Nevertheless, this KG focuses primarily on ingredient-disease associations and lacks structured modeling of complex medicinal diet objects [8]. TCM-KLLaMA incorporates a knowledge injection mechanism of fuzzy matching of synonyms, which can to some extent suppress the model’s hallucination phenomenon [9]. KG‑based question-answering systems and medicinal diet question-answering systems have also demonstrated the potential of integrating KGs with LLMs, but none have systematically addressed the critical issues of safety constraints and evidence traceability in medicinal diet recommendations [10–12]. In summary, existing intelligent medicinal diet recommendation systems still exhibit clear deficiencies in interpretability and safety.

The integration of KGs, RAG, and LLMs provides a novel technical approach to tackle these challenges. KGs can structure the relationships among medicinal diets, ingredients, effects, and contraindications, making recommendation results traceable to specific original information and thereby enhancing the interpretability. Dense vector retrieval can precisely align colloquial queries with standard terminology through semantic embeddings, effectively bridging the terminology gap. RAG encapsulates retrieved evidence as context information, constraining LLM output through evidence-based reasoning and reducing the occurrence of model hallucinations. Moreover, a safety-first principle can be systematically incorporated into the recommendation ranking and generation via evidence-constrained supervised fine-tuning [7–9].

In response to the progress and limitations of existing work, we constructed and evaluated a graph-semantic hybrid retrieval and evidence-constrained explainable generation system for medicinal diet recommendations, named ShenNongDiet. This system is built upon the *Guoyi Jinghua Yao Shan* book series and includes a Neo4j KG containing 3376 medicinal diets along with multiple entity and relationship types. It integrates core modules, including entity linking, a dual-path retrieval strategy combining symbolic (KG) and dense (vector) retrieval, weighted fusion ranking, evidence encapsulation, and LoRA-based supervised fine-tuning.

Using a dedicated evaluation set ShenNongDiet-Test and multiple baseline ablation experiments, we assessed the system’s performance in terms of retrieval accuracy, generation faithfulness, and evidence constraints adherence. The aim is to provide a technological framework with traceable evidence for medicinal diet recommendations in consumer health informatics and clinical decision support systems.

## Methods

### Overview of ShenNongDiet recommendation system

**Fig. 1:**
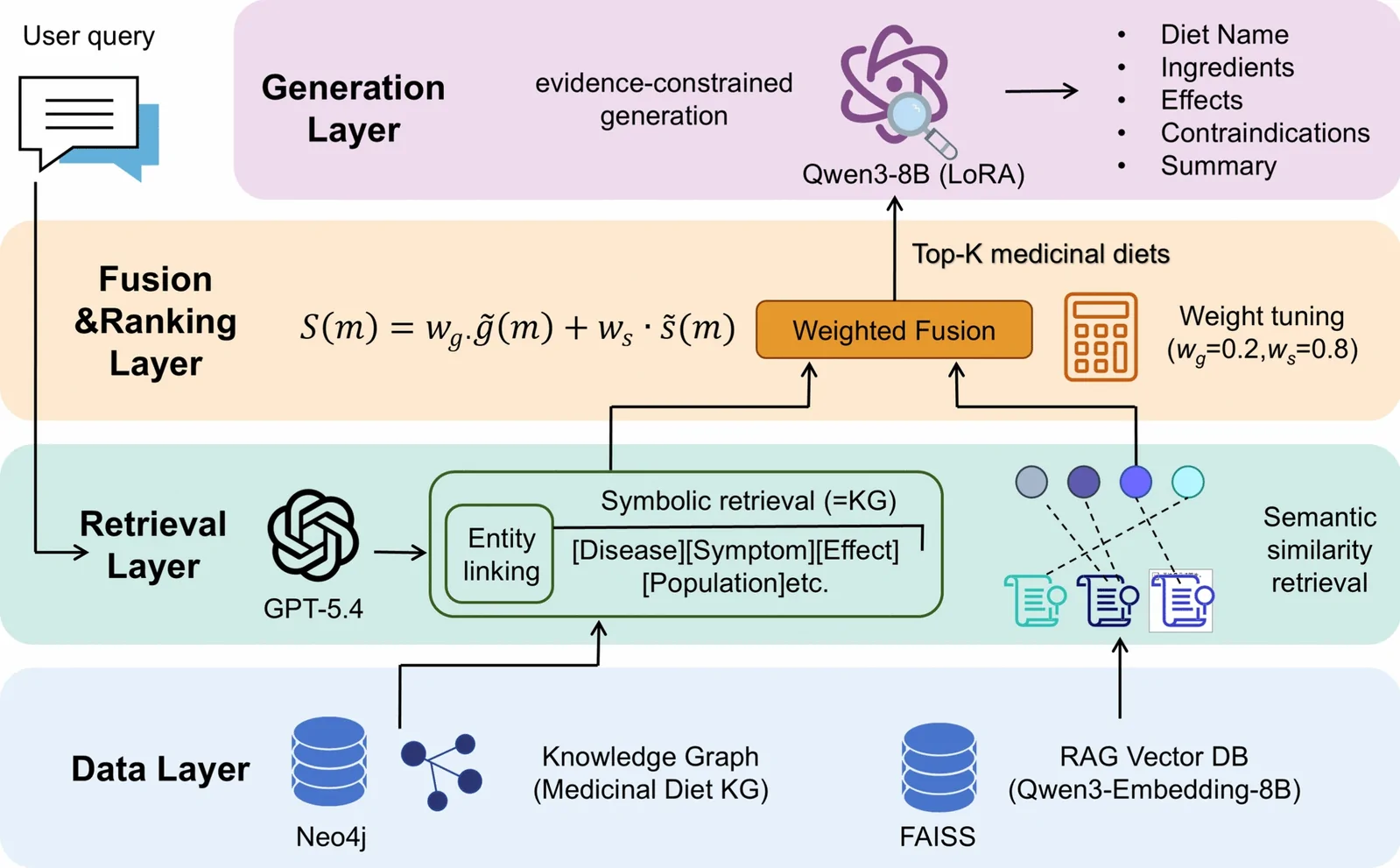
Overall architecture of ShenNongDiet. The system processes a user query through two parallel pathways: (1) symbolic retrieval from a Neo4j KG of medicinal diets; (2) dense vector retrieval from a FAISS‑indexed RAG database using Qwen3‑Embedding‑8B. The top‑15 results are then passed to an evidence‑constrained generation module (Qwen3‑8B with LoRA) to produce a structured output including diet name, ingredients, effects, contraindications, and a summary

The overall architecture of the intelligent medicinal diet recommendation system mainly includes four modules: KG, retrieval enhancement, rule weighting, and fine-tuning model, as shown in Fig. 1. Based on the user’s natural language description q, the system simultaneously conducts KG query and vector semantic retrieval. The KG query uses an OpenAI GPT-5.4 model to parse the user’s intent in q (disease, symptom, effect, population, or constitution, etc.), and retrieves the KG of medicinal diets based on the parsed entity and their types. The model was selected for this study because of its capabilities in natural language understanding, multi-condition entity extraction, and structured output generation. It was accessed remotely through a Chat Completions API compatible with the OpenAI API specification, using the gateway routing identifier openai/gpt-5.4. To improve the reproducibility of the study, the same system prompt, user-query template, predefined JSON fields, and post-processing procedures were used throughout the experiments. The decoding parameters were set to temperature = 0.1 and max_tokens = 2048. To avoid missing retrieval results due to terms in q that are not covered by the KG, and to preserve the complete semantic meaning of the user query as much as possible, vector semantic retrieval is performed to encode q into a high-dimensional vector and retrieve the top-50 candidate results from the text vector database of medicinal diet. Subsequently, the system merges the results from KG retrieval and semantic retrieval. A rule-based weighting module assigns weights of 0.2 and 0.8 to the KG channel and the semantic retrieval channel, respectively, and computes a fused score for each candidate medicinal diet. The top-15 ranked items along with their associated evidence are then selected as the context for the generation model, which is the LoRA-fine-tuned Qwen3-8B, to generate the final top-5 recommendations and their corresponding explanations. The entire workflow is implemented using locally deployed Dify 1.13.0 (https://github.com/langgenius/dify/).

### Data source

The medicinal diet data used in this study were sourced from the book series *Guoyi Jinghua Yao Shan* [13–21]. After undergoing Optical Character Recognition (OCR), 3385 pieces of unstructured medicinal diet data were obtained. Based on a comprehensive comparison across all fields-including the medicinal diet name, formula, preparation, efficacy, consumption method, and remarks-8 groups of completely duplicate records and 1 group of records with equivalent content but different field ordering were merged, yielding a final dataset of 3376 medicinal diet records. These data primarily cover seven categories: (1) Medicinal diet name (full name, e.g., stir-fried Chinese cabbage with coix seed); (2) Formulation (ingredients and their dosages, e.g., coix seed 15 g, Chinese cabbage 300 g); (3) Production steps (e.g., grinding, cutting, cooking order); (4) Efficacy (therapeutic descriptions, e.g., disinfecting and draining dampness, invigorating the spleen and clearing damp-heat); (5) Administration method (method of intake, e.g., once daily, can be served as a side dish); (6) Notes (nature, flavor and meridian tropism of certain ingredients, e.g., Chinese cabbage is slightly cold in nature, sweet in flavor, neutral in tropism; and acts on the large intestine and stomach meridians; contraindications and suitable populations for the medicinal diet, e.g., contraindicated for constipation; suitable for alopecia); and (7) source (bibliographic information, e.g., *Guoyi Jinghua Yao Shan: A Complete Guide to Beauty and Skincare Medicinal Diets*). These fields serve not only as the input for KG extraction and standardization but also as the basic units of the subsequent medicinal diet text corpus.

### Dataset construction

To clarify the specific purposes of each data resource, based on the 3376 medicinal diet records, we constructed the following five data resources after three specialists performed data cleaning, standardized symbol representation, and removed entries with missing information:

(1) Medicinal diet text vector database. The medicinal diet data were organized into structured textual corpora according to six fields: name, formula, preparation steps, efficacy, consumption method, and remarks. Each text was encoded into a 4,096-dimensional vector using Qwen3-Embedding-8B, forming a database with a 3376×4096 dimensional vector matrix. This database primarily serves the semantic completion function, mainly compensating for the insufficiency of explicit relations in the KG and providing a more comprehensive evidence candidate set for subsequent RAG generation.

(2) Base model evaluation dataset. Based on the medicinal diet data fields (formula, efficacy, and applicable symptoms), we constructed 2000 single-choice questions, with easy and hard questions set at a 6:4 ratio. The easy questions are “formula-to-efficacy” tasks, requiring the selection of the correct main efficacy from four options based on the given medicinal ingredients; the stem and correct answer are derived from the formula and corresponding efficacy fields, while distractors are selected from similar efficacy descriptions of other medicinal diets. The hard questions are “efficacy/symptom-to-formula” tasks, requiring the correct formula to be identified from multiple options with similar or partially overlapping ingredients; the stem and correct answer are derived from the efficacy/applicable symptom fields and their corresponding formula fields, while distractors are constructed through controlled operations such as ingredient deletion, replacement, or addition to the correct formula. Each question has a unique correct answer with a clear original textual basis. After generation, researchers reviewed each question for stem‑record consistency, answer traceability and uniqueness, semantic overlap among options, distractor rationality, and absence of information beyond the original corpus.

(3) LoRA supervised fine-tuning dataset. Based on the medicinal diet text and KG data, using the DeepSeek-V4-Pro LLM in a human–machine collaborative manner, 9,377 LoRA supervised fine-tuning samples were constructed. The construction process involved: standardizing original texts and removing invalid records; validating field completeness and JSON format of generated samples; verifying evidence traceability of responses (ensuring that key entities and attributes are locatable with no unsupported information); and manual spot-checking for text-KG consistency, response completeness, and safety constraint expression. Samples failing these checks were revised or excluded before inclusion in the fine-tuning dataset. The supervised fine-tuning data were randomly split at a ratio of approximately 9:1 into a training set of 8509 samples and a validation set of 868 samples, with the training set used for LoRA parameter updates and the validation set used solely for performance monitoring and model selection during training. The dataset primarily covers tasks such as medicinal diet recommendation, single-diet explanation, comparison of different diets, and comprehensive synthesis of multiple pieces of evidence.

The supervised fine-tuning corpus follows the “Instruction-Context-Response” three-element format compatible with LLaMA-Factory:

Instruction: Covers the major generation tasks encountered in real-world system application, including: recommending medicinal diets based on diseases, symptoms, or health needs; explaining the composition, efficacy, preparation method, applicable conditions, and contraindications of a single medicinal diet; comparing the characteristics of different medicinal diets for the same disease or symptom; and providing comprehensive explanations based on multiple text excerpts and KG relations. All tasks require the model to respond strictly based on the provided evidence.

Context: Summarizes the textual descriptions of medicinal diets (e.g., name, formula, preparation steps, efficacy, usage, contraindications, and target population) as well as KG relation summaries (e.g., alleviates diseases, contraindicated populations). All contexts are derived from curated and verified research corpora, strictly excluding unverified external information.

Response: Extracts or maps relevant fields from the evidence context according to deterministic rules or templates. For recommendation tasks that require system invocation, the output adopts a structured JSON format; for explanatory tasks, the output adopts a structured natural language format. Information not explicitly stated in the evidence is uniformly labeled as “not recorded in the provided materials,” with no external knowledge augmentation. Key content (medicinal diet names, ingredients, efficacy, target populations, and contraindications) must have corresponding evidence in the context; generation of efficacy, compatibility, or contraindication information without evidentiary support is prohibited.

(4) System evaluation test set. Based on medicinal diet texts and KG data not used for model fine-tuning, 150 natural language question-answering (QA) pairs were constructed via semi-automatic machine generation assisted by manual verification. The scenarios cover single symptom (16.7%), multiple syndrome types (14.0%), multiple symptoms (41.3%), multiple diseases (9.3%), special populations (16.0%), and contraindication QA (2.7%); representative examples are provided in Supplementary Table S1. Reference answers were obtained by screening candidate medicinal diets from the texts and KG based on the diseases, symptoms, syndrome types, efficacy, ingredients/preparation forms, applicable populations, and contraindication conditions contained in the questions, and were then verified by tracing back to the original corpus and KG relations. Each question retains 1–3 medicinal diets that satisfy all constraints as reference answers. The test questions simultaneously cover colloquial symptom descriptions, TCM terminology, ingredient/preparation preferences, and population restrictions. Special population scenarios include children, adolescents, pregnancy, postpartum, and middle-aged/elderly groups. Contraindication QA scenarios are designed to evaluate the system’s ability to identify unsuitable, contraindicated, and avoidable medicinal diet relations. This test set is used for final system performance evaluation and related comparative experiments.

(5) Weighting evaluation test set. Using the same method as the system evaluation test set, 100 non-overlapping QA pairs were constructed, with 1–3 standard medicinal diet names annotated as reference answers for each question. The scenarios are primarily multi-symptom queries, while also covering single symptom, multiple syndrome types, multiple diseases, and special populations.

### KG module

Based on the top-down KG construction method and entity and relationship types from the previous study [12], the design of the pattern layer in this research follows the basic principles of domain ontology modeling, which constrains knowledge through core concepts and their semantic relationships. The modeling boundary is set by the evidence chain required for medicinal diet recommendations, and the entities and their relationships are organized around “medicinal diet-ingredient-effect-diseases or symptoms-applicable and contraindicated populations”. The entity extraction and normalization of the KG are completed through a human–computer collaboration approach. The DeepSeek-V4-Pro LLM is used to extract candidate entities in batches according to the pre-defined 6 types of entity categories under a fixed prompt template (Table 1).

**Table 1:** Types of KG entities and examples

Entity type	Example
Medicinal diet	shouwu stir-fried liver and kidney
Disease	hypertension
Ingredient	processed shouwu (12 g)
Symptom	soreness and weakness of waist and knees
Effect	nourishing liver and kidney
Population	people with hypertension of kidney-yin deficiency type
	people with loose stools or damp-phlegm constitution

During the entity normalization process, the “*Chinese Pharmacopoeia*”, “*Chinese Food Composition Table*”, “*Chinese Materia Medica*”, “*The Basic Theory of Chinese Medicine*” [22–26] and other structured data that have been pre-organized as standard terminology libraries are used as the standard terminology database. The candidate entities are deduplicated, matched, and screened within the same entity type, and the directly matched ones are mapped to the standardized names. Eight researchers combined the formulas, efficacy, and remarks fields in the original records to verify the unmatched synonyms, variant names, and ambiguous terms one by one; entries that could not be determined or had differences were reviewed by three domain experts. Synonyms are merged only when it is confirmed that they point to the same concept; variant names are standardized only when the writing differences do not change the entity identity; ambiguous terms are disambiguated based on the entity type, source field, and context. Non-dietary medicine entities are merged only when the type, standardized name, and semantic are the same; dietary medicine entities are jointly compared in terms of name, formula, preparation method, usage, and source, and the name is not used as the sole basis for deduplication. The mapping between the original name and the standardized name is retained during the normalization process; entries with insufficient evidence retain the original text, and no mapping or relationship is established.

Ingredients and their dosages were parsed from the “formulation” field to establish the “COMPOSED_OF” relation between medicinal diets and ingredients. Entities of effect, diseases, and symptoms were extracted from descriptions of “efficacy”, and the relations of “HAS_EFFECT”, “ALLEVIATES”, and “MODULATES” were established respectively. Suitable and contraindicated populations were identified from the “notes” field to construct corresponding relations such as “SUITABLE_FOR” and “CONTRAINDICATED_FOR” between medicinal diets and population groups (Table 2). Finally, the verified entity and relationship data were imported into the Neo4j graph database to construct a medicinal diet KG.

**Table 2:** Types of entity relationships in KG and examples

Entity relationship type	Example	Example related entities	Definition
COMPOSED_OF	<shouwu stir-fried liver and kidney, COMPOSED_OF, processed shouwu (12 g)>	Medicinal diet, Ingredient	Identifies an ingredient and its dose in a medicinal diet
HAS_EFFECT	<shouwu stir-fried liver and kidney, HAS_EFFECT, nourishing liver and kidney>	Medicinal diet, Effect	Links a medicinal diet to a documented traditional dietary function
MODULATES	<shouwu stir-fried liver and kidney, MODULATES, soreness and weakness of waist and knees>	Medicinal diet, Symptom	Links a medicinal diet to a symptom it may regulate or improve
ALLEVIATES	<shouwu stir-fried liver and kidney, ALLEVIATES, hypertension>	Medicinal diet, Disease	Links a medicinal diet to a disease whose impact or burden it may alleviate; it does not denote treatment
SUITABLE_FOR	<shouwu stir-fried liver and kidney, SUITABLE_FOR, people with hypertension of kidney-yin deficiency type>	Medicinal diet, Population	Identifies a population for whom the medicinal diet is considered suitable
CONTRAINDICATED_FOR	<shouwu stir-fried liver and kidney, CONTRAINDICATED_FOR, people with loose stools or damp-phlegm constitution>	Medicinal diet, Population	Identifies a population for whom the medicinal diet is unsuitable or requires caution

After identifying the user's intention based on OpenAI GPT-5.4, the graph entity identifiers for diseases, symptoms, effects, etc. were obtained. The system then retrieved candidate medicinal diet data based on relationships such as “ALLEVIATES”, “MODULATES”, “HAS_EFFECT”, and matched them by having the disease, symptom, and effect nodes point to the medicinal diet nodes.

For each candidate medicinal diet, the number of hits that were consistent with the entities mentioned by the user in the above relationship types was counted (for example, if the user mentioned multiple symptoms, this medicinal diet would be counted once for each symptom where there was a “modulate” edge). Then, the sum of the hit numbers of each type was calculated to obtain the score related to the graph of this candidate. Since this score is composed of countable edge-level hits, it can directly correspond to specific relationships and entities, serving as the basis for user display, and thus ensures interpretability.

###  Retrieval enhancement module

Based on the medicinal diet text vector corpus, a FAISS IndexFlatL2 exact vector index was constructed, and Euclidean distance was employed for similarity retrieval. Meanwhile, vector identifiers corresponding one-to-one to the medicinal diet nodes in the KG were established for subsequent semantic retrieval and the fusion of dual-path recall results. The user query is converted into the same-dimensional query vector through Qwen3-Embedding-8B, and the top 50 candidate medicinal dishes are retrieved from the index. The Euclidean distance is mapped to a bounded similarity score:

$$s=\frac{1}{1+d}$$

As the semantic channel score of this candidate. This channel is used to retrieve medicinal diets that are not explicitly associated as edges in the graph but are similar to the user’s description in natural language, complementing symbolic retrieval.

### Base model evaluation

Based on the “base model evaluation dataset”, under the unified prompt engineering and model parameter conditions (temperature = 0, top_p = 1), compare the understanding capabilities of Qwen3-8B, Hunyuan-7B, Baichuan2-7B, glm-4v-9b and DeepSeek-R1-8B for traditional Chinese dietary knowledge, and select the base model for subsequent LoRA fine-tuning.

the accuracy rate of “understanding the efficacy of medicinal diet” (Acc_efficacy_) was used to evaluate the model’s semantic understanding ability of the corresponding efficacy and applicable scenarios of medicinal diet, the accuracy rate of “understanding the recipe of medicinal diet” (Acc_ingredient_) was used to evaluate the model’s cognitive understanding of the composition and the rules of medicinal ingredient combination, and the TCM Understanding Index (TUI) was calculated to comprehensively evaluate the overall understanding level of the model in the field of traditional Chinese medicinal diet:

$$TUI=0.594\times {Acc}_{efficacy}+0.406\times {Acc}_{ingredient}$$

The open-source model with the highest TUI score was selected as the base model for LoRA fine-tuning in this study. This strategy ensures that the selected model already has strong prior knowledge of TCM, which helps to reduce the hallucination risk during the fine-tuning process and improve sample efficiency.

### Model fine-tuning module

To enhance the consistency between the model’s generated results and the retrieved evidence, and to reduce the generation of unsupported content, this study conducted evidence-constrained supervised fine-tuning of the optimal base model Qwen3-8B in LLaMA-Factory based on the LoRA supervised fine-tuning dataset. LoRA can control training costs and maintain iterative efficiency by only updating low-rank adaptation parameters [27] (Table 3). After the training is completed, the fine-tuned model will be deployed to the local inference service to generate the recommendations for medicinal diets and their evidence explanations.

**Table 3:** Key configurations for LoRA supervised fine-tuning

Category	Configuration item	Value
Base Model & Framework	Base Model	Qwen3-8B
Base Model & Framework	Training Framework	LLaMA-Factory
LoRA Architecture	finetuning type	lora
LoRA Architecture	lora rank (r)	128
LoRA Architecture	lora alpha (α)	256
LoRA Architecture	lora target	all
Input & Template	template	qwen3
Input & Template	cutoff len	4096
Optimizer & Scheduler	learning rate	5×10⁻^5^
Optimizer & Scheduler	lr scheduler	cosine
Optimizer & Scheduler	warmup ratio	0.05
Optimizer & Scheduler	weight decay	0.01
Training Batch	per-device train batch size	4
Training Batch	gradient accumulation steps	2
Training Batch	effective batch size	8
Training Precision & Epochs	num train epochs	3
Training Precision & Epochs	precision	bf16
Validation Settings	eval strategy	steps
Validation Settings	eval steps	200
Validation Settings	per-device eval batch size	2
Dataset	train dataset	tcmhe_rag_sft_train
Dataset	eval dataset	tcmhe_rag_sft_eval
Dataset	samples (train/eval)	8509/868

To evaluate the impact of LoRA fine-tuning on the model’s generation performance, a comparative experiment was conducted between the Qwen3-8B model and the LoRA fine-tuned model based on the system evaluation test set. For the same test query, the two models received exactly the same frozen KG retrieval evidence and vector retrieval evidence in terms of the content and order. The fusion weights of KG retrieval and vector retrieval were both fixed at (0.2, 0.8), and during the evaluation process, only the generation model was replaced to control the influence of the retrieval result differences on the comparison results. The evaluation indicators included Hit@1, Hit@3, average answer recall rate, and evidence traceability ratio.

### Rule-weighted module

After merging the candidate medicinal diets obtained from the dual-path recall (KG retrieval and semantic vector retrieval), each candidate needs to be uniformly ranked. Let the current batch of candidate medicinal diets set be *C*. For each candidate *m∈C*, denote its original score output by the KG module as *g(m)*, and the original similarity score output by the semantic vector retrieval module as *s(m)* (for example, based on cosine similarity or distance mapping). To eliminate the influence of different channel score scales, perform maximum normalization on the two types of scores within the set *C* separately:

$$\widetilde{g}\left(m\right)=\frac{g(m)}{{max}_{m^{\prime}\in c}g({m}^{{\prime}})}$$

$$\widetilde{s}\left(m\right)=\frac{s(m)}{{max}_{m^{\prime}\in c}s({m}^{{\prime}})}$$

The values of *g̃(m)* and *s̃(m)* both fall within the range of [0, 1]. Ultimately, the fusion ranking score *S(m)* of the candidate medicinal diets m is defined as:

$$S\left(m\right)={w}_{g\cdot }\widetilde{g}\left(m\right)+{w}_{s}\cdot \widetilde{s}(m)$$

Here, ${w}_{g}$,${w}_{s}$ represent the graph evidence weight and the semantic similarity weight respectively, and they satisfy ${w}_{g}+{w}_{s}=1$ to ensure that the fusion score maintains the same scale. This study aims to strike a balance between the interpretability of the KG and the recall rate of semantic vectors. Under the condition that other factors remain unchanged, nine parameter combinations were compared based on the weighted evaluation of the test set to obtain the optimal weight selection. Here, “${w}_{g}$” ∈ [0.1, 0.9] and “${w}_{s}$” ∈ [0.9, 0.1]. The evaluation indicators used were Hit@1 and Mean Reciprocal Rank (MRR), and the 95% confidence intervals of the two indicators were calculated using the bootstrap method.

### System Evaluation

To evaluate the effectiveness and robustness of ShenNongDiet in the natural language-based herbal cuisine question-answering scenario, an assessment was conducted based on the system evaluation test set under the same hardware environment (NVIDIA RTX PRO 6000 96 GB, Ubuntu 22.04, CUDA 12.8). The 95% confidence intervals of Hit@K and MRR were evaluated using the bootstrap method, and the significance of the differences in hits between systems was tested using the paired McNemar test.

### Precision retrieval evaluation

For each query task, the system returns a list of medicinal diets ranked by score in the top K positions. If there is a standard answer, it is considered a hit. The core performance indicators for evaluation are Hit@K and MRR.

### RAGAs evaluation

In this study, the RAGAs framework (version 0.4.3) was used to evaluate the contribution of the RAG module to the generation of medicinal diet recommendations. Faithfulness was used to determine whether the generated answers were supported by the retrieved evidence on medicinal diet. Context recall was used to assess whether the information required to generate the answers was covered by the retrieved context. Context precision was used to evaluate whether relevant contexts were ranked ahead of irrelevant contexts. These three metrics respectively reflect the degree of evidential support for the generated content, as well as the coverage and relevance of the retrieved context, and can be used to quantitatively evaluate the system’s retrieval and generation performance.

### Human assessment indicators

Two experts with over 3 years of clinical experience in TCM independently and blindly rated the Top-K medicinal diets returned by ShenNongDiet and their explanations in four aspects: accuracy, top-5 recommendation set repeatability, relevance, and faithfulness. The accuracy of the system was labeled as binary. When the explanation was consistent with the evidence and the standard answer, it was marked as 1; otherwise, it was marked as 0. Top-5 recommendation set repeatability was the proportion of pairwise comparisons among top-5 sets from three independent runs in which experts judged at least four recommendations to share semantic overlap in medicinal diets, main effects, population constraints, and evidence conclusions. Possible scores were 0, 1/3, 2/3, or 1. When the quoted fragment could be used to answer the user’s question, it was marked as relevant; otherwise, it was marked as irrelevant. When the key statements in the answer could be found with clear evidence in the quoted evidence and there was no directional conflict, it was marked as faithful; otherwise, it was marked as not faithful. Let N be the total number of samples quoted evidence, where N_r is the number of relevant evidence and N_f is the number of faithful evidence that supported the key statements. Then, the relevance score Rel is defined as N_r/N and the faithfulness score Fai as N_f/N. When N = 0, this sample is marked as missing and excluded from the main statistics.

### Ablation and comparative experiments

In this study, graph-only (using only the KG channel for retrieval and ranking without using vector semantic retrieval), dense-only (using only vector retrieval without using the graph channel), and Best Match 25 (BM25) (using only sparse retrieval based on term matching) were selected as the baselines for the comparative experiments. These experiments compared the performance differences among symbolic retrieval, semantic retrieval, and hybrid retrieval. At the same time, the independent contributions of each sub-module to ShenNongDiet were analyzed.

## Results

### Intelligent search interface for medicinal diet

**Fig. 2:**
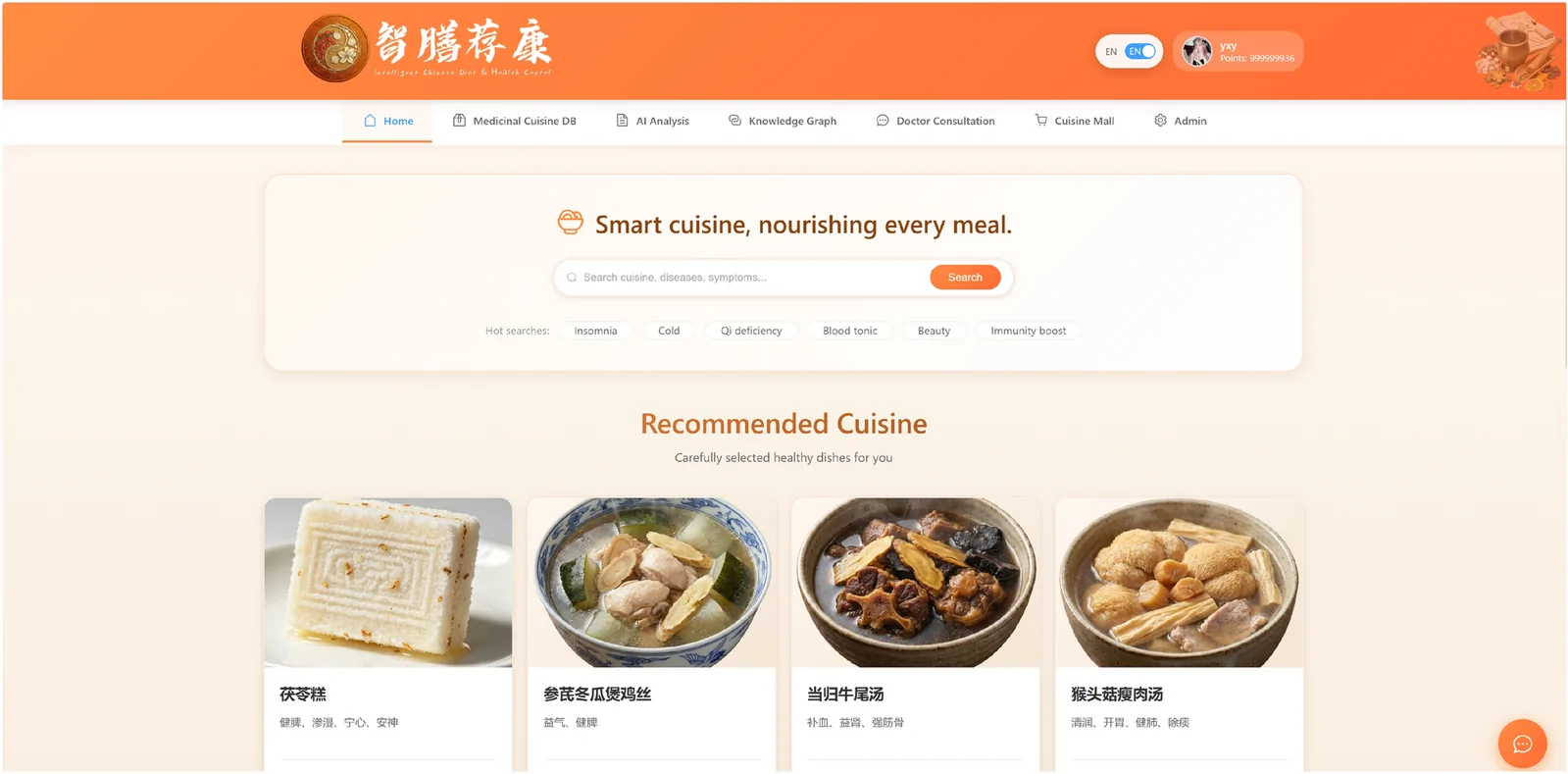
Main user interface of ShenNongDiet

**Fig. 3:**
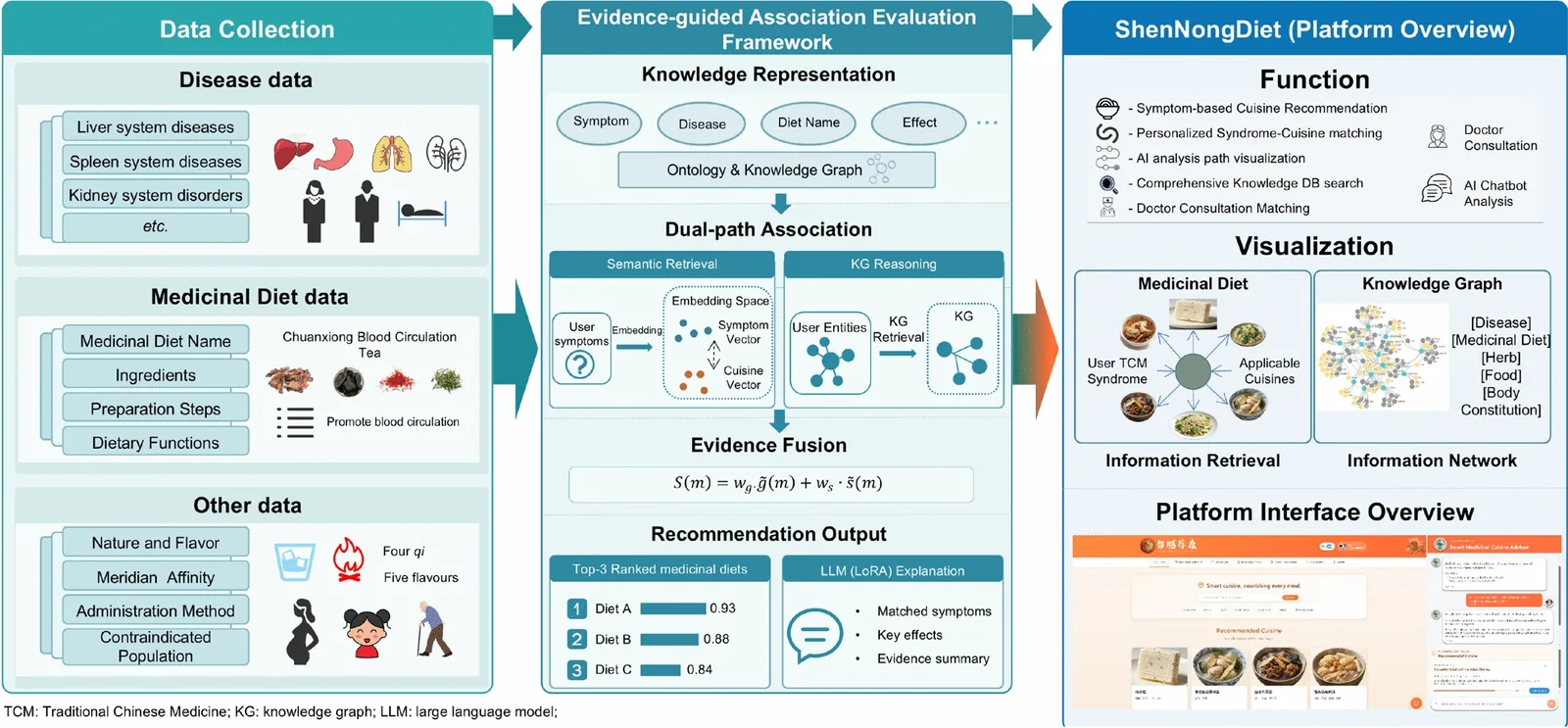
Protocols for the data collection, the association evaluation, and the overview of the ShenNongDiet platform

We have developed a public-oriented portal for intelligent recommendation services of medicinal diet, accessible via computer browsers (Fig. 2). The system is built on a KG of TCM and TCM text, using the RAG framework. While ensuring the accuracy and traceability of medical information, the system provides three core functions: natural language question answering with text and voice input, personalized recommendations based on users’ constitutions and health goals, and contraindication alerts for special populations or contraindicated conditions (Fig. 3).

**Fig. 4:**
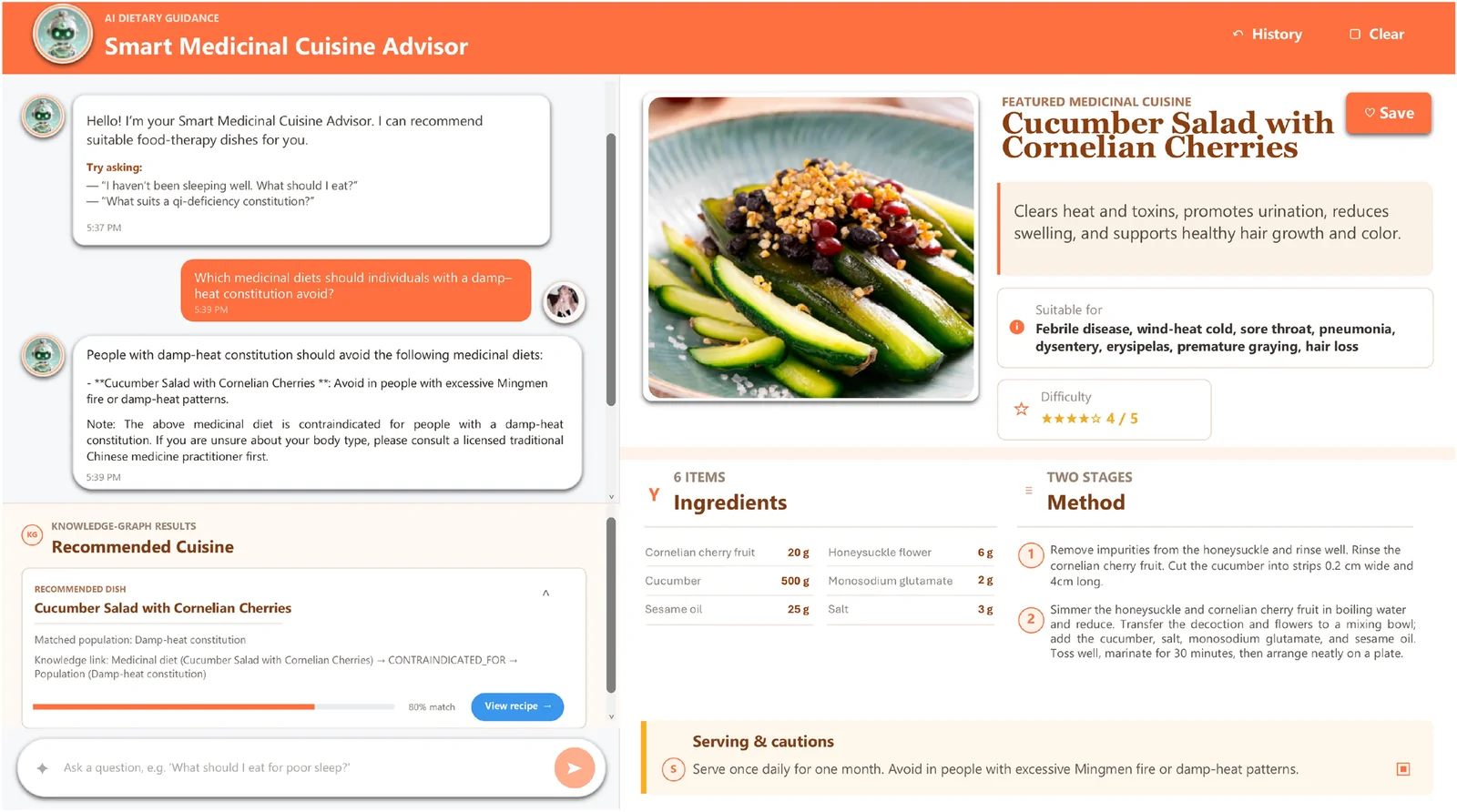
Examples of questions and dietary taboos in the user interface of the ShenNongDiet system

The system adopts an evidence priority strategy to suppress the hallucinations that LLMs tend to produce in vertical medical scenarios. For example, when a user inputs “Which medicinal diets should individuals with a damp-heat constitution avoid?”, the system first performs entity recognition and graph search, then combines semantic recall to generate a structured answer. The response includes the recommendation or contraindication conclusion, a traceable evidence path based on knowledge graph relations, matching scores, related symptoms and diseases, as well as information on ingredients, preparation methods, and precautions for consumption. Taking Cucumber Salad with Cornelian Cherry officinalis as an example, when the query matches a contraindication rule associated with a damp-heat constitution, the system issues a corresponding warning and displays the KG relation path “Medicinal diet-CONTRAINDICATED_FOR-Population”. The recipe details section simultaneously presents the dish’s general indications and consumption precautions, thereby clearly specifying its applicable conditions and conditional contraindications (Fig. 4). If the knowledge base lacks direct evidence, the system clearly indicates “There is currently insufficient evidence in the database”, only providing a risk-controlled reference explanation and refusing unsupported assertions. While improving the usability of the question-answering system, this mechanism helps enhance the transparency of information sources, the traceability of evidence, and the auditability of safety constraints; however, it cannot replace professional clinical judgment.

### Basic data of the KG

**Fig. 5:**
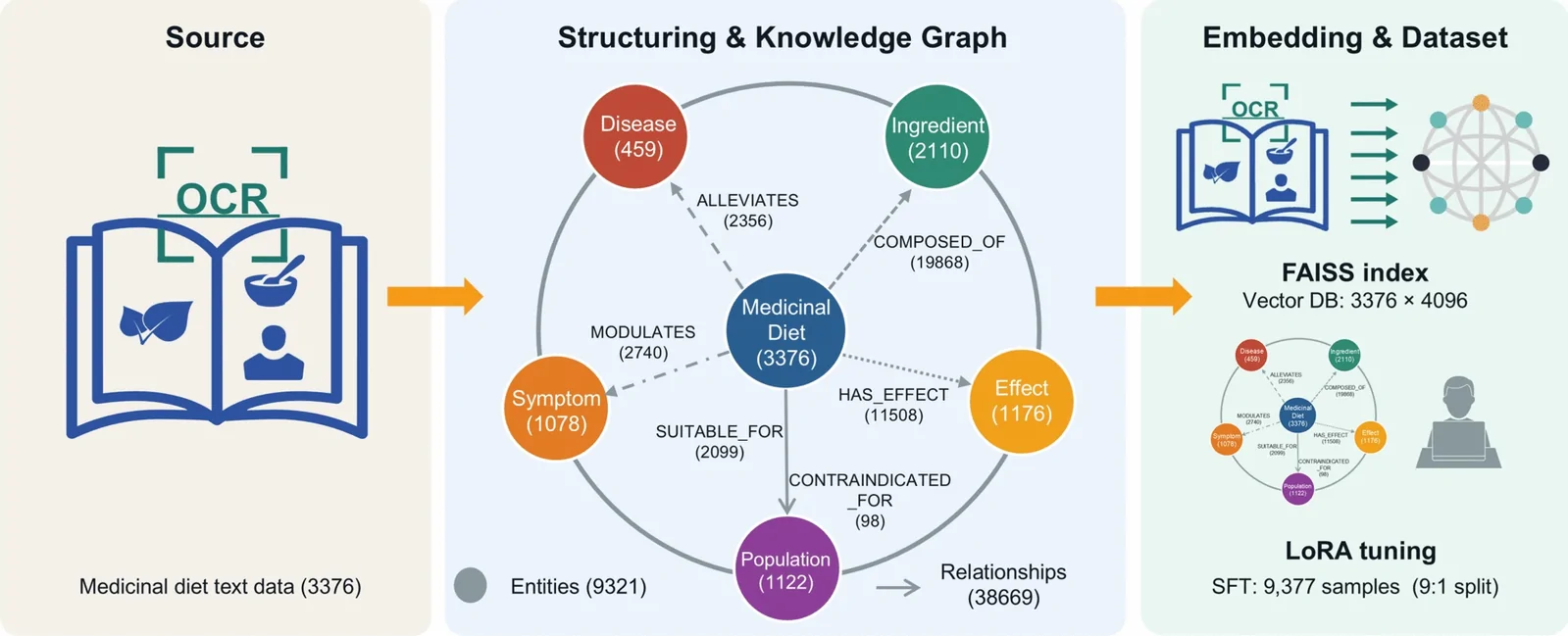
Source of data support for the ShenNongDiet system

Through systematic sorting and standardization, structured data were extracted from the *Guoyi Jinghua Yao Shan* book series (Fig. 5). A total of 9321 entities were extracted, including 3376 medicinal diet entities, 2110 ingredient entities, 1176 effect entities, 1122 population entities, 1078 symptom entities, and 459 disease entities. In addition, 38,669 relations were constructed, including 19,868 COMPOSED_OF, 11,508 HAS_EFFECT, 2740 MODULATES, 2356 ALLEVIATES, 2099 SUITABLE_FOR, and 98 CONTRAINDICATED_FOR relations.

### Base model evaluation results

**Fig. 6:**
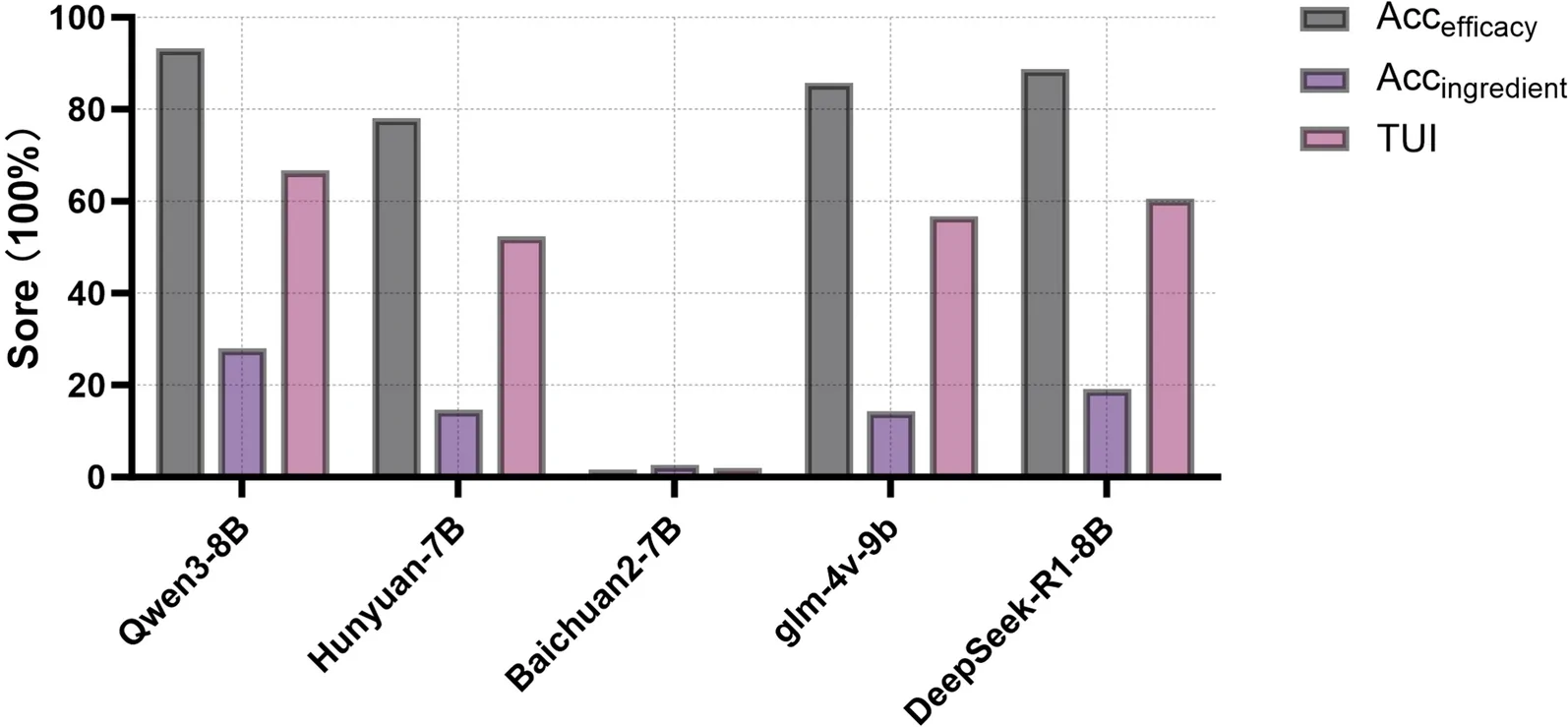
Performance comparison chart of different LLMs on three evaluation indicators

The evaluation results show that the Qwen3-8B model achieved Acc_efficacy_ of 93.27%, Acc_ingredient_ of 27.96%, and TUI of 66.75% (Fig. 6, Table 4). It outperformed other participating models in both efficacy understanding and recipe understanding tasks. The understanding capabilities of DeepSeek-R1-8B, glm-4v-9b, and Hunyuan-7B models were slightly weaker, with TUI values of 60.50%, 56.75%, and 52.30% respectively. They all had a common weakness in recipe understanding ability, with the highest Acc_ingredient_ being only 19.09%. Baichuan2-7B performed poorly overall, with a TUI of only 2.05%, unable to meet the basic requirements of the TCM diet understanding task. The results indicate that current open-source LLMs generally have insufficient structured cognition of medicinal diet knowledge. However, Qwen3-8B demonstrates relatively better understanding ability in this field and can serve as the base model for ShenNongDiet architecture.

**Table 4:** Comparison results among base models

Model	Acc_efficacy_ (%)	Acc_ingredient_ (%)	TUI (%)
Qwen3-8B	93.27	27.96	66.75
Hunyuan-7B	78.03	14.66	52.30
Baichuan2-7B	1.60	2.71	2.05
glm-4v-9b	85.77	14.29	56.75
DeepSeek-R1-8B	88.80	19.09	60.50

### Comparison results before and after model fine-tuning

Under the same retrieval evidence and fusion weight conditions, the overall performance of the LoRA fine-tuned model is superior to that of the base Qwen3-8B model. The Hit@1 and Hit@3 of the base model are 69.9% and 80.4% respectively, and after LoRA fine-tuning, they increase to 74.8% and 82.5% respectively. The average recall rate of answers increases from 44.44% to 56.89%, indicating that the fine-tuned model can generate more correct medicinal diet results consistent with the reference answers. The proportion of evidence traceability increases from 58.67% to 84.67%, indicating that LoRA fine-tuning enhances the model’s ability to generate answers based on retrieval evidence and cite relevant evidence. Overall, the evidence-constrained LoRA supervised fine-tuning improves the coverage of correct answers and the ability to cite evidence.

### Integration of weight selection results

On 100 weight search samples, 9 sets of complementary weights that satisfy ${w}_{g}+{w}_{s}=1$ were compared (Table 5). When $\left({w}_{g},{w}_{s}\right)=(\mathrm{0.2,0.8})$, the Hit@1 reached 84.00% (95% CI: 77.00%−89.00%), and the MRR reached 0.8865 (95% CI 0.8276–0.9283). The point estimates of both indicators were the highest among the 9 configurations. The suboptimal configuration (0.1, 0.9) had a Hit@1 of 78.00% (95% CI 70.00–85.00%) and an MRR of 0.8494 (95% CI 0.7929–0.9072). When the graph weight increased to 0.4 or above, both indicators significantly decreased, indicating that the candidate ranking of this task mainly relies on semantic retrieval, and retaining a smaller graph weight is helpful for integrating structured evidence. Therefore, the system adopts (0.2, 0.8) as the default integration weight.

**Table 5:** Comparison of retrieval performance for different fusion weight combinations

w_g_	w_s_	Hit@1(%)	MRR
0.1	0.9	78.00 [70.00, 85.00]	0.8494 [0.7929, 0.9072]
0.2	0.8	84.00 [77.00, 89.00]	0.8865 [0.8276, 0.9283]
0.3	0.7	68.00 [56.00, 76.00]	0.7925 [0.7138, 0.8524]
0.4	0.6	44.00 [34.00, 53.00]	0.6527 [0.5811, 0.7115]
0.5	0.5	23.00 [14.00, 32.00]	0.4679 [0.3853, 0.5344]
0.6	0.4	4.00 [1.00, 8.00]	0.1951 [0.1558, 0.2393]
0.7	0.3	3.00 [0.00, 7.00]	0.1500 [0.1119, 0.1944]
0.8	0.2	2.00 [0.00, 5.00]	0.1153 [0.0844, 0.1530]
0.9	0.1	3.00 [0.00, 6.00]	0.1230 [0.0863, 0.1618]

### Accuracy of retrieval evaluation results

This study adopts a hybrid framework of graph retrieval and semantic retrieval. The evaluation results are shown in Table 6. The system has a stable advantage in Top-K retrieval: Hit@1 is 84.00%, Hit@3 is 90.00%, Hit@5 is 92.67%, MRR is 0.8778, and the 95% confidence interval width of the bootstrap method is narrower, indicating that the performance estimation of the system has good accuracy.

**Table 6:** Main results table (point estimates + bootstrap 95% confidence intervals)

Metric	Point estimate	95% CI
Hit@1 (%)	84.00	[77.33, 88.67]
Hit@3 (%)	90.00	[85.33, 94.00]
Hit@5 (%)	92.67	[87.33, 96.00]
MRR	0.8778	[0.8288, 0.9260]

### RAGAs evaluation results

In order to further rigorously evaluate the role of the RAG module in actual QA scenarios, the RAGAs framework was adopted for the assessment. The evaluation results showed that the faithfulness was 0.831, indicating that most of the medicinal diet recommendations could be supported by the retrieved evidence; the context recall was 0.526, suggesting that the key information required for the generated results could be covered by the retrieved context; however, the context precision was only 0.064, indicating that the relevant context was not adequately concentrated and prioritized throughout the set of retrieved evidence, and there were still many redundant or insufficiently relevant contents in the retrieval results. This shows that the RAG module has achieved certain effects in providing evidence support for the generated content, which can reduce the hallucination phenomenon in the generation of LLMs to a certain extent; however, the quality of the screening and sorting of the retrieved evidence still has obvious limitations. Although the retrieval strategy prioritizes comprehensiveness, it inevitably introduces redundant noise data, thereby reducing the density of relevant evidence. Future optimization of the re-ranking algorithm is needed.

### Human evaluation indicators assessment results

To rigorously evaluate ShenNongDiet, two experts in TCM independently conducted blinded assessments of 150 recommendation sets across four dimensions (Table 7). This system has a certain degree of accuracy in recommending medicinal diets. The average scores of the two experts were 0.807 ± 0.396 (Expert 1) and 0.920 ± 0.272 (Expert 2), indicating that the majority of the recommended medicinal diets were consistent with the gold standard of the system’s evaluation test set. The consistency of the top-5 recommendation set repeatability was 0.215 ± 0.173 and 0.218 ± 0.164, respectively. A score of 0.215 indicates that, on average, slightly more than one‑third of the three pairwise comparisons met the ≥ 4/5 semantic overlap criterion, reflecting moderate output variation across repeated runs rather than low recommendation accuracy or low evidence faithfulness. Under the strict criterion of “at least 4/5 overlap”, these scores were at a relatively low level, indicating that there were certain changes in the top-5 recommended medicinal diets during repeated runs, which to some extent reflects the diversity of the recommendation results, but not for evaluating the recommendation accuracy, evidence fidelity, or clinical reliability. The relevance scores were 0.894 ± 0.215 and 0.933 ± 0.197, indicating that most of the referenced fragments returned by the system can be directly used to answer the corresponding user questions, and the retrieved evidence has a high semantic relevance to the query content. The faithfulness scores of the two experts were 0.651 ± 0.301 and 0.648 ± 0.283, indicating that the explanations of the generated results were generally consistent with the referenced evidence. These results show that ShenNongDiet performs well in terms of recommendation accuracy, evidence relevance, and generation fidelity, demonstrating its reliability as an evidence-driven medicinal diet recommendation tool.

**Table 7:** Results of human expert ratings

Metric	Expert 1 (Mean±SD)	Expert 2 (Mean±SD)	Expert 1 (Median [IQR])	Expert 2 (Median [IQR])
Accuracy	0.807 ± 0.396	0.920 ± 0.272	1.000 [1.000, 1.000]	1.000 [1.000, 1.000]
Top-5 recommendation set repeatability	0.215 ± 0.173	0.218 ± 0.164	0.333 [0.000, 0.333]	0.333 [0.000, 0.333]
Relevance	0.894 ± 0.215	0.933 ± 0.197	1.000 [0.867, 1.000]	1.000 [1.000, 1.000]
Faithfulness	0.651 ± 0.301	0.648 ± 0.283	0.733 [0.400, 0.917]	0.667 [0.400, 0.933]

**Fig. 7:**
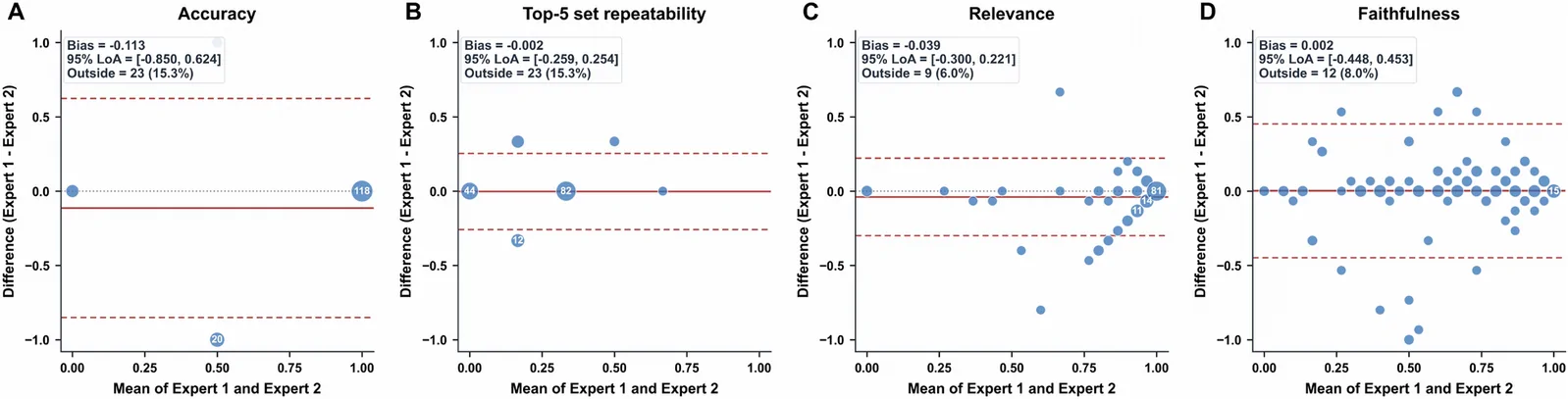
Bland–Altman consistency analysis of two traditional Chinese medicine experts in four evaluation dimensions. **A** Accuracy; **B** Repeatability consistency of the top-5 recommended sets; **C** Relevance; **D** Faithfulness. Each indicator includes 150 pairs of scores. The red solid line represents the average difference of the two experts’ ratings, the red dotted line represents the 95% consistency limit, and the gray dotted line represents a difference of 0. The bubble area represents the number of overlapping observations

To evaluate the consistency of the scores given by the two domain experts, we used the Bland–Altman analysis method to conduct quantitative evaluation from four dimensions: accuracy, top-5 recommendation set repeatability, correlation, and fidelity. As shown in Fig. 7, the average differences of the four indicators are all close to 0. Among them, the top-5 recommendation set repeatability indicator LoA is the narrowest ([− 0.259, 0.254]) and all sample points are within the range, indicating the best consistency; the accuracy ([− 0.850, 0.624]) and correlation ([− 0.300, 0.221]) indicators have most sample points within the range, indicating good consistency; the fidelity LoA has the largest span ([− 0.448, 0.453]), and a few samples exceed the boundaries, indicating relatively higher subjective differences. There is no obvious funnel-shaped distribution in each figure, and the score differences do not show a regular pattern with the mean value. Overall, the two experts showed good agreement in their ratings across the four dimensions, indicating that the expert evaluation data were reliable.

### Results of ablation and comparison experiments

Based on the ablation experiment results of 150 valid use cases (Table 8), the proposed hybrid framework in this study performed optimally across all evaluation metrics. Its MRR and Hit@1 were significantly higher than any single retrieval channel (*P* < 0.001), further demonstrating the effectiveness of the complementary strategy that integrates symbolic KG retrieval with dense semantic retrieval for medicinal diet retrieval. As shown in the experiments, the dense-only channel achieves far better performance than the graph-only mode in the complex semantic environment of medicinal diet question-answering tasks, since the latter is inherently constrained by path coverage. The core advantage of the hybrid system lies in the structured constraints provided by multi-path fusion, which significantly optimizes the accuracy of the top result (Top-1); although the difference in the Hit@5 dimension between it and dense-only decreases due to semantic coverage saturation as K increases (*P* = 0.2188), its performance gain in precise reordering still establishes the leading position of the hybrid architecture.

**Table 8:** Ablation study results of ShenNongDiet

Method	MRR	Hit@1 (%)	Hit@3 (%)	Hit@5 (%)
ShenNongDiet	0.8748	84.0[78.0, 89.3]	89.3[84.0, 94.0]	92.0[87.3, 96.0]
Graph only	0.3936	29.3[22.0, 37.3]^***^	40.0[32.0, 48.0]^***^	47.3[39.3, 56.0]^***^
Dense only	0.7360	61.3[53.3, 68.7]^***^	84.0[77.3, 90.0]^*^	89.3[84.0, 94.0]
BM25 only	0.5897	48.0[40.0, 56.7]^***^	64.7[56.7, 72.0]^***^	70.0[62.7, 77.3]^***^

*P < 0.05, **P < 0.01, ***P < 0.001; compared with ShenNongDiet

## Discussion

In this study, we proposed and validated ShenNongDiet, an intelligent recommendation system for medicinal diets that integrates a KG, dual‑path retrieval (symbolic and semantic), and rule‑weighted fusion. The system aims to address the growing demand for evidence-based and personalized recommendations in the field of medicine-food homology. Unlike prior work that focuses on formula generation or general medicinal diet suggestions, ShenNongDiet is the first to deeply couple symbolic reasoning from a structured KGs with dense-vector semantic understanding, and to significantly enhance interpretability via evidence-constrained supervised fine-tuning. This provides a new paradigm for intelligent medicinal diet applications.

Our results further confirm that the hybrid retrieval framework substantially outperforms any single retrieval channel in Top-1 accuracy (84.0% vs. 29.3%–61.3%), mainly due to the complementary strengths of symbolic and semantic retrieval. The symbolic graph channel provides interpretable evidence anchors through explicit relations such as “MODULATES”, “ALLEVIATES” and “HAS_EFFECT”, but its recall is limited when the user’s expression does not match the graph terms; pure dense-vector retrieval (dense-only) can expand the coverage through semantic generalization, but it cannot provide structured evidence traceability and is prone to noise interference in rare medicinal diet scenarios. Compared to the recent work of Sha et al., Yaoshi-RAG (Hit@1 = 84.2%) [8], ShenNongDiet achieves a higher MRR (0.8778) while maintaining a comparable hit rate, demonstrating the advantage of hybrid architecture in precise re-ranking.

This study primarily adopted an evidence-first generation strategy, enabling medicinal diet recommendations and their accompanying explanations to be traced back to relevant records in the knowledge base. After LoRA fine-tuning, the proportion of evidence traceability increased from 58.67% to 84.67%, indicating that fine-tuning enhanced the model’s ability to retrieve evidence and generate answers, and did not generate prohibited hallucinatory content. In the RAGAs evaluation, the faithfulness was 0.831 and the context recall was 0.526, suggesting that the RAG module helps to enhance the consistency between the generated content and the retrieved evidence; moreover, the median accuracy rate evaluated by TCM experts was 1.000, indicating that the system output has a high degree of consistency with the experts’ judgment of the reference answers. The above results jointly support the effectiveness of the system under the current testing conditions from three aspects: ranking hit rate, evidence fidelity, and expert judgment.

However, the evaluation results also revealed several areas requiring further optimization. First, the RAGAs context precision score was only 0.064, while the MRR reaches 0.8748. This discrepancy primarily resulted from the use of a relatively broad evidence window, comprising approximately 15 context passages, to maintain adequate recall. The recall‑oriented design ensures that truly relevant evidence is rarely missed in complex queries with multiple symptoms or constraints; however, it also resulted in the retrieved context containing substantial irrelevant and redundant background information, thereby reducing the density of useful evidence. Future research will introduce a two-stage Cross-encoder re-ranker to retain the wide range of full recall capability and perform refined re-ranking filtering on the retrieval results, compressing redundant noise and purifying effective context, thereby further improving retrieval accuracy and information utilization. Secondly, the consistency of the top-5 recommendation set repeatability is relatively low, indicating that the output results of the same query vary in different runs. This may be related to the diversity of reasonable candidate medicinal diets, the changes in the ranking of similar candidates, and the randomness of generation. We will improve this by implementing sorting calibration and random control in the future. Finally, there are only 98 instances of the “CONTRAINDICATED_FOR” relationship in the KG, mainly derived from the explicit prohibited or cautious use information recorded in the existing medicinal diet corpora. It is still difficult to cover all risk scenarios for different diseases, constitutions, and special populations. Therefore, the current results only indicate that the system has the ability to conduct contraindication search and constraints based on existing evidence. It cannot yet prove that it has a comprehensive and reliable risk identification capability or clinically validated safety. In the future, we will integrate authoritative sources such as pharmacopoeias, clinical guidelines, and professional medicinal diet literature, expand the contraindication knowledge, and have experts verify it. At the same time, we will construct a special safety test set to further evaluate the recall rate of contraindication identification, the rate of inappropriate recommendations, and the accuracy of rejection.

Further, as with most comparable studies, our knowledge base construction relies on a limited number of high-quality, expert-reviewed data sources. While this approach minimizes the risk of information distortion, it may also restrict the coverage of rare or complex diseases. Reasoning over uncertain KGs allows for the incorporation of knowledge on a larger scale and in the presence of noise, or serves as a powerful supplement to expand the boundaries of the system. In the future, we aim to incorporate multi-dimensional diagnostic information to build a more comprehensive and three-dimensional “diagnosis and treatment” intelligent agent.

## Conclusions

This study developed and validated ShenNongDiet, an innovative system that integrates a KG, a hybrid dual-path retrieval mechanism (semantic vectors and symbolic logic), and LoRA-fine-tuned LLMs under evidence constraints. In recommendation scenarios, the system effectively reduces the risk of generating unsupported content through an evidence-constrained mechanism and provides personalized medicinal diet recommendations that are interpretable, traceable, and in line with the principles of TCM diagnosis. By explicitly modeling constitution suitability, ingredient contraindications, and evidence‑bound generation, ShenNongDiet provides a reference implementation path for incorporating safety constraints in intelligent diet recommendation systems. Beyond lowering the barrier for users to access professional medicinal diet guidance, this work lays a solid methodological foundation for future clinical decision support and real-world data-driven personalized health management.

## Supplementary Information

Supplementary material 1.

## Data Availability

Data are provided within the published article and its online supplementary material.

## Abbreviations

BM25: Best Match 25
KG: Knowledge graph
LLM: Large language model
LoRA: Low-Rank Adaptation
MRR: Mean Reciprocal Rank
NATCM: National Administration of Traditional Chinese Medicine
OCR: Optical Character Recognition
QA: Question-answering
RAG: Retrieval-augmented generation
SFT: Supervised fine-tuning
TCM: Traditional Chinese medicine
TUI: Traditional Chinese Medicine Understanding Index
WHO: World Health Organization
