# Supplementary material for: ShenNongDiet: a medicine diet recommendation system based on graph-semantic hybrid retrieval and evidence constraint generation
Source: Chin Med. 2026 Aug 26;21:214. doi: 10.1186/s13020-026-01505-x (PMC13508355; doi:10.1186/s13020-026-01505-x)
Supplement: Supplementary file 1 [file 13020_2026_1505_MOESM1_ESM.docx]

**Supplementary Materials**

**Table S1. Composition and representative examples of the system evaluation test set**

**Table S1. Composition and representative examples of the system evaluation test set**

| **Questiontype** | **Number(n)** | **Proportion(%)** | **Representative question** | **Corresponding reference answer(s)** |
| --- | --- | --- | --- | --- |
| Single-symptom query | 25 | 16.7% | 我晚上看东西不清楚，还有夜盲症，想问问吃什么主食能帮助改善视力？（I have poor night vision and night blindness. Which staple-food medicinal diets may help improve vision?） | 青葙子猪肝卷；猪肝绿豆粥；玄参杞麦蛋面（Celosia Seed and Pork Liver Rolls; Pork Liver and Mung Bean Congee; Scrophularia–Goji–Ophiopogon Egg Noodles） |
| Multiple TCM-pattern query | 21 | 14.0% | 针对不同情况导致的酒糟鼻，比如热毒很重、长丘疹时热毒湿气都重、或者血瘀凝结的那种，有没有对应的药膳？（For rosacea associated with different TCM patterns—such as intense heat toxin, papular lesions with concurrent heat toxin and dampness, or congealed blood stasis—are there corresponding medicinal diets?） | 三黄白菜包；马齿苋双花饼；桃仁白茅根饮（Sanhuang Chinese Cabbage Bun; Purslane and Two-Flower Cake; Peach Kernel and Cogongrass Rhizome Drink） |
| Multiple-symptom query | 62 | 41.3% | 更年期综合征，中医说是肝郁化火那种类型，主要症状是失眠，还特别口渴总想喝水，眼睛发红、嘴里发苦，小便黄、大便干结，有这些上火表现，适合吃什么药膳？（For menopausal syndrome characterized by liver constraint transforming into fire, with insomnia, marked thirst, red eyes, a bitter taste, yellow urine, and constipation, which medicinal diets are suitable?） | 柴胡决明子粥；柴胡决明粥；栀子仁粥（Bupleurum and Cassia Seed Congee; Bupleurum and Cassia Congee; Gardenia Seed Congee） |
| Multiple-disease query | 14 | 9.3% | 我有心血管病或者糖尿病这些慢性病，中医说是气阴两虚的体质，想找一些能日常调理、帮助延年益寿的药膳，最好是像茶饮这样简单方便的形式，有吗？（I have chronic conditions such as cardiovascular disease or diabetes and a TCM pattern of dual qi–yin deficiency. Are there simple medicinal diets, preferably tea preparations, for daily regulation and health maintenance?） | 西洋参黄芪茶；红参党参茶；益寿排骨汤（American Ginseng and Astragalus Tea; Red Ginseng and Codonopsis Tea; Longevity Pork Rib Soup） |
| Special-population query | 24 | 16.0% | 怀孕期间失眠、心情烦躁、容易抑郁，想找一些能养心安神、疏解郁闷的药膳，最好是做起来简单的茶或者粥，有推荐吗？（During pregnancy, I have insomnia, irritability, and low mood. Are there simple medicinal diets, preferably teas or congees, that nourish the heart, calm the spirit, and relieve emotional constraint?） | 养心粥；枣仁安神粥；绿萼梅安神（Heart-Nourishing Congee; Sour Jujube Seed Calming Congee; Green-calyx Plum Blossom Calming Tea） |
| Contraindication/avoidance query | 4 | 2.7% | 湿热体质的人，有哪些药膳是不能吃、需要避免的？（Which medicinal diets should individuals with a damp–heat constitution avoid?） | 山茱萸拌黄瓜（Cucumber Salad with Cornelian Cherry） |
| Total | 150 | 100.0% | \ | \ |
